# High Consumption of Soft Drinks Is Associated with an Increased Risk of Fracture: A 7-Year Follow-Up Study

**DOI:** 10.3390/nu12020530

**Published:** 2020-02-19

**Authors:** Li Chen, Ruiyi Liu, Yong Zhao, Zumin Shi

**Affiliations:** 1 School of Public Health and Management, Chongqing Medical University, Chongqing 400016, China; nameclx@foxmail.com (L.C.); lry981118@foxmail.com (R.L.); 2 Research Center for Medicine and Social Development, Chongqing Medical University, Chongqing 400016, China; 3 The Innovation Center for Social Risk Governance in Health, Chongqing Medical University, Chongqing 400016, China; 4 Chongqing Key Laboratory of Child Nutrition and Health, Chongqing 400016, China; 5 Human Nutrition Department, College of Health Science, QU Health, Qatar University, Doha 2713, Qatar; zumin@qu.edu.qa

**Keywords:** soft drinks, fracture, longitudinal study, epidemiology, China Health and Nutrition Survey

## Abstract

(1) Background: Fracture causes a substantial burden to society globally. Some studies have found that soft drinks consumption was associated with the risk of fractures. We aimed to assess the association in the Chinese population; (2) Methods: Data from 17,383 adults aged 20 to 75 years old attending the China Health and Nutrition Survey (CHNS) between 2004 and 2011 were analyzed. Soft drinks consumption and fracture occurrence were self-reported. The cross-sectional and longitudinal associations between soft drink and fracture was assessed using multivariable mixed-effect logistic regression and Cox regression; (3) Results: After adjusting for sociodemographic and lifestyle factors and dietary patterns, compared with those who did not consume soft drinks, participants with daily consumption of soft drinks had an odds ratio (95%CI) of 2.72 (95%CI: 1.45–5.09) for fracture. During a mean 5-year follow-up, there were 569 incident fracture cases. Compared with non-consumers, those with daily soft drinks consumption had a hazard ratio (95%CI) of 4.69 (95%CI: 2.80–7.88) for incident fracture; (4) Conclusions: Soft drinks consumption is directly associated with the risk of fracture. Reducing soft drinks consumption should be considered as an important strategy for individual and population levels to maintain bone health.

## 1. Introduction

Musculoskeletal diseases such as fractures pose a serious global burden of disease. Based on the Global Burden of Disease study, musculoskeletal illness accounted for 6.8% of total disability-adjusted life-years worldwide in 2010 [1]. In China, it has been estimated that more than 4 million people suffered from traumatic fractures in 2014 [2]. The average total medical cost due to fracture is estimated to be Renminbi (RMB) 18,853 per year per patient in China [3]. Within the next 40 years, individuals at high risk of osteoporosis fractures (i.e., aged 50 years or older) will place a significant burden on society, particularly in Asia [4].

Although the direct causes of fractures are accidental falls or hits, the fundamental causes are low bone density and excessive bone loss [5,6]. Unhealthy lifestyle behaviors are associated with fractures [7]. Diet is an important determinant of bone health. The role of calcium, dairy products and vitamin D in bone health has been widely reported [8,9]. Moreover, diets rich in fruits and vegetables provide high levels of trace elements and vitamins can increase bone density and reduce the risk of fractures [10]. While the role of protein on bone health is still inconclusive, studies on the overall dietary pattern and fracture have attracted attention [11].

During the past three decades, China’s urbanization has transformed the traditional dietary pattern into a modern dietary pattern [12]. Accompanied by the dietary transition, the burden of chronic diseases increased. A study using China Health and Nutrition Survey (CHNS) data suggested a direct association between modern dietary pattern (characterized by a high intake of energy-dense and processed food) and fracture [11]. However, the associations between individual food items in the modern diet and fracture have not been well studied. One of the important components of the modern diet is soft drinks. In recent years, the consumption of soft drinks in China has been on the rise, especially among young people [13]. The high consumption of soft drinks increases the risk of obesity, diabetes and other chronic non-communicable diseases (NCDs) [14]. Excessive consumption of soft drinks can also reduce the intake of healthy drinks such as milk, leading to a lower intake of trace elements such as calcium and magnesium, which can increase the risk of osteoporosis and fracture [15,16]. 

Most of the existing studies on soft drinks consumption have focused on bone mineral density, (BMD) with few studies on fracture [17]. Several studies have focused on the effects of carbonated beverages on adolescent fractures [18]. Drinking large amounts of carbonated beverages during the development of adolescents may reduce the accumulation of bone minerals and increase the risk of future fractures [19]. A high content of phosphoric acid in soft drinks has been hypothesized to be one of the mechanisms linking soft drinks and fracture. Excessive intake of phosphoric acid changes calcium/phosphorus ratio and imbalance of not only the calcium and phosphorus ratio but also the acid-base in the body, resulting in decreased bone density and even osteoporosis and fractures [20,21,22].

Soft drinks consumption is positively associated with obesity risk. Obesity is a risk factor for fractures in specific bone sites and a prospective cohort study which involving 17 sites in 10 countries found that obesity is a risk factor for upper arm/shoulder and clavicle fractures [23,24]. However, no studies have assessed the association between soft drinks and fracture in the Chinese population. We aimed to use the China Health and Nutrition Survey (CHNS) to assess the prospective association between habitual soft drinks consumption and fracture risk in Chinese adults.

## 2. Materials and Methods 

### 2.1. Study Design and Population

**Figure 1:**
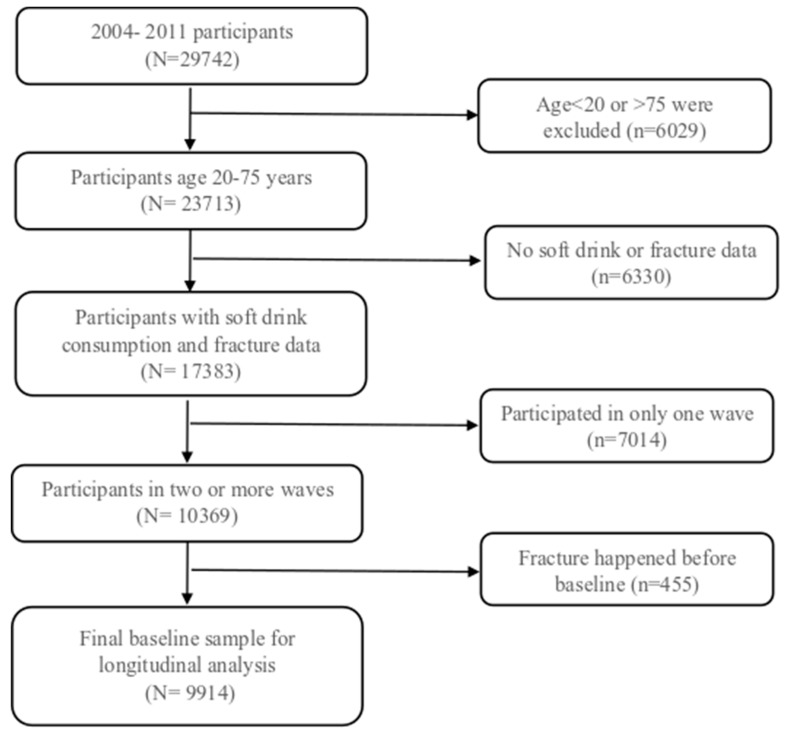
Sample flowchart.

CHNS is an open prospective cohort study which was conducted in nine provinces in China [25]. Ten waves of surveys were conducted from 1989 to 2015. Participants may join or leave at any wave of the surveys. A multistage random-cluster sampling technique was used to select households in this study. All members of the selected families were invited to partake in the study. As the frequent consumption of soft drinks was assessed only in 2004, 2006, 2009 and 2011 surveys, the main analysis of the current study used data from the surveys between 2004 and 2011. Between 2004 and 2011, 29,742 participants joined the study at least once. After excluding participants who were not eligible (age below 20 or above 75 years (people with older age may suffer from chronic diseases which may be confounding factors of fracture), had no information of soft drinks (N = 6325) or fracture (N = 5)), a total of 17,383 participants were included in the analysis (Figure 1). In the longitudinal analysis of those without fracture at baseline, the sample size was 9914. This study was approved by the National Institute of Nutrition and Food Safety (Beijing, China) and the institutional review committees of the University of North Carolina (Chapel Hill, NC, USA). All the participants signed informed consent before the survey.

### 2.2. Outcome Variable

Data of fracture were self-reported in each wave in 2004, 2006, 2009 and 2011 by the question “Have you ever had a fracture?” along with age when the first fracture occurred [11]. In the analysis, the incident fracture was defined if a participant reported a new history of fracture.

### 2.3. Soft Drinks Consumption

Soft drinks consumption was determined in 2004, 2006, 2009 and 2011 by the question “Last year, did you drink soft drinks or sugared fruit drinks?”. When the participants answer “yes” to this question, they were asked to provide more information of what types of soft drinks ((1) Chinese brand soft drinks; (2) Non-Chinese brand soft drinks; (3) Sugared fruit drinks) and the frequency of the consumption: (1) almost every day; (2) 3–4 times/week; (3) 1–2 times/week; (4) 1–2 time/month; (5) no more than once a month; (9) unknown. In order to distinguish the difference between “unknown” and other answers of questions in the original questionnaire, when the answer is “unknown”, the “unknown” coded as “9”. Chinese and non-Chinese brands include carbonated drinks, sugar-sweetened dairy drinks, etc. The soft drinks consumption frequency was categorized into five levels: non-consumers, <1 time/week, 1–2 times/week, 3–4 times/week, and almost daily. The participants were also asked about the amount of each type of soft drinks (liter) consumption each week. We calculated the total amount of soft drinks consumption and categorized into three categories: (1) non-consumers; (2) <1 L/week; (3) ≥1 L/week.

### 2.4. Covariates

Socio-demographic, lifestyle, physical measurements and chronic conditions data were collected at each wave. The highest level of education achieved was divided into three groups: low (illiterate or primary school), medium (junior middle school) and high (high middle school or higher). The residency was divided into three urbanization levels based on an urbanization index which is a composite of 12 components that included population and other socioeconomic characteristics [12]. Lifestyle factors included in the analysis were smoking (non-smoker, former smokers, and current smoker), alcohol consumption (yes or no) and physical activity levels (PAL). PAL in terms of metabolic activities of the task (MET-hours per week), was calculated based on self-reported job and leisure time activities, intensity and duration of the activities. Weight in kilograms divided by height in meters squared was used to calculate the Body Mass Index (BMI). Detailed dietary consumption was assessed using 3-day food records in each survey wave. Intake of energy, fat, protein, carbohydrate, phosphorus, and calcium was calculated using the Chinese Food Composition Table. To reflect the overall dietary intake, we constructed dietary patterns using a factor analysis. Food intake was first divided into 35 groups before conducting the factor analysis. The following criteria were used to determine the number of factors: (1) Eigenvalue (>1.5); (2) scree plot, and (3) interpretability of the factors was used to determine the number of dietary patterns. Factor loadings and percentages of variances were calculated. Factor scores were assigned across all study participants for each dietary pattern. To reduce correlation between patterns and facilitate the interpretability, factor scores were orthogonally (varimax) rotated. Two dietary patterns were identified, including modern dietary pattern and traditional southern dietary pattern based on the previous study [11,26]. The traditional south pattern is characterized by a high intake of rice, vegetables, and pork, and a low intake of wheat; a modern dietary pattern is characterized by a high intake of fruit, milk, egg, soy milk, and deep-fried products. In the sensitivity analyses, we excluded soft drink consumption while constructing dietary patterns in order to control the potential over adjustment in the multivariable analysis.

Hypertension was determined based on diastolic (above 90mmHg) and/or systolic (above 140 mmHg) blood pressure measures or having doctor-diagnosed hypertension. Diabetes was determined based on having doctor-diagnosed diabetes.

### 2.5. Statistical Analysis

To compare differences between groups for categorical variables, the chi-square test was used and ANOVA was used for continuous variables. Mixed effect logistic regression was used to assess the association between soft drinks consumption and the prevalence of fracture. A set of models was used: model 1 adjusted for age and sex; Model 2 further adjusted for intake of energy and fat, smoking, alcohol drinking, income, urbanization, education, physical activity, and BMI; model 3 further adjusted for two dietary patterns (traditional south pattern and modern pattern; model 4 further excluded those participated only in wave of survey. We did not consider missing values a problem as the survey used a face-to-face interview method. The full multivariable mixed effect model included 15,956 participants, representing 91.8% of the original analytical sample. We also assessed the association between soft drinks consumption and incident fracture using Cox regression. Cox proportional hazards assumptions were investigated by visual inspection of log-log plots generated by stphplot syntax in Stata, showing no deviations.

All the analyses were performed using STATA 15.1 (Stata Corporation, College Station, TX, USA). Statistical significance was considered when *p* < 0.05 (two-sided).

## 3. Results

In general, there was a negative correlation between soft drinks consumption and age in 2004 (Table 1). Men were more likely to drink soft drinks than women. Soft drinks consumption was directly associated with urbanization, education, smoking and fat intake. Among those attended in all four surveys (n = 4423), the prevalence of soft drinks consumption was 22.4%, 18.6%, 30.1% and 33.1% in 2004, 2006, 2009, and 2011, respectively. 

Soft drinks consumption was directly associated with fracture after adjusting for potential confounding factors. There was a dose-response direct relationship between soft drinks consumption and fracture. The odds ratios (95%CI) across soft drinks consumption levels of none, <1 time/week, 1–2 times/week, 3–4 times/week and almost daily were 1.00, 1.16 (0.94–1.44), 1.32 (0.95–1.84), 1.70 (0.99–2.92), and 2.72 (1.45–5.09) (*p* for trend <0.001), respectively (Table 2). 

Among the 9914 participants free of fracture at baseline, 569 developed fracture during 49,838 person-year follow-up. The mean follow-up duration was 5.0 (SD 2.1) years. Compared with non-consumers of soft drinks, daily consumers had a hazard ratio of 4.69 (95%CI 2.80–7.88) for fracture after adjusting for sociodemographic and lifestyle factors including overall dietary patterns. However, the actual consumption of soft drinks did not show a statistically significant association with the fracture. Compared with non-consumers, those who consumed ≥1 L/week had an HR of 1.16 (95% CI 0.83–1.61) for fracture (Table 3).

In the sensitivity analyses, when we adjusted for dietary patterns without soft drinks, the above associations between soft drinks and fracture remained

## 4. Discussion

A high consumption of soft drinks was associated with an increased risk of fracture in the current large prospective cohort study. Daily consumption of soft drinks was associated with a doubled risk of fracture independent of sociodemographic and lifestyle factors as well as overall dietary patterns.

### 4.1. Comparison with Other Studies

Despite the large number of studies on the association between soft drinks and non-communicable diseases, studies on soft drinks and fracture among adults are limited [27,28,29]. To the best of our knowledge, this is the first study on soft drinks and fracture in the Chinese population. Our finding of a direct association between soft drinks consumption and fracture is in line with the existing studies in the USA [27,28]. In the Nurse Health Study, among postmenopausal women, each additional serving of total soda per day was associated with a 14% increased risk of hip fracture (RR: 1.14; 95% CI: 1.06, 1.23) [27]. Another study which included 161,808 postmenopausal women found that modest increased risk of hip fracture was associated with high soda consumption. [30] In the cross-sectional study conducted in the USA, among women former college athletes, nonalcoholic carbonated beverage consumption had a odds ratio for fracture of 2.28 (95% CI 1.36, 3.84) [28]. Several cross-sectional studies conducted among children and adolescents also found a direct association between soft drinks and fracture [18,19]. 

Our findings have public health significance. The prevalence of soft drinks consumption in the 2004 survey of this study was 24%, which is lower than in the USA. However, there has been a rapid increase in soft drinks consumption in China. Data from the 2010–2012 national survey suggest that the prevalence of soft drinks consumption reached 50% [31]. The contribution of soft drinks to fracture may increase in the future. Intervention to reduce soft drinks consumption is needed. As age is a strong determinant of soft drink consumption, it is important for the government take measures to reduce soft drink consumption in the younger generation in order to prevent the potential adverse effects of soft drinks on bone health.

### 4.2. Potential Mechanisms

Several mechanisms may explain our findings on the direct association between consumption of soft drinks and fracture. The Chinese population may be at a high risk of fracture due to a low intake of calcium. Calcium intake was below 400 mg/d in all the soft drinks consumption groups. Soft drink contributes to the dietary intake of phosphorus. High phosphorus but low calcium diet may stimulate parathyroid hormone and cause bone resorption [29]. A high intake of phosphorus may also reduce the renal activation of 25-hydroxyvitamin D and affects calcium homeostasis [32]. In our study, no difference was found in the intake of phosphorus by soft drinks consumption levels. Overall, the intake of phosphorus in the Chinese diet is high. The addition of phosphorus from soft drinks may not contribute much due to relatively low consumption. However, the absorption of inorganic phosphorus may be higher than organic phosphorus from other foods. 

Certain ingredients in soft drinks can affect bones. Sugar and sodium in soft drinks can increase the loss of calcium [33,34]. Increased consumption of caffeinated beverages also increases the risk of fractures [35] and recurrent fractures [36]. It should be noted that tea is also a protective factor for fractures, increasing bone mineral density [37]. The tea drink prevalence is high in the Chinese population. In a Chinese prospective study of half a million people, drinking tea daily reduced the risk of hip fracture [38]. Another study conducted in Singapore showed that drinking four cups of coffee daily increased hip fracture risk [39]. The comparison between soft drinks consumers with non-consumer is in a way comparing soft drinks and tea in the Chinese context. 

Another possible mechanism for the indirect effect of soft drinks on bone fractures is the mediating effect of obesity. It has been shown in many studies that the consumption of soft drinks increases the obesity risk [40]. Fat affects the regulation of bone and is involved in the bone active hormones metabolism [41,42]. Increased fat content in muscle leads to more falls, which can increase the fracture risk in specific areas [43,44]. People with obesity lose their normal protective mechanisms and are more inclined to fall backward or sideways than forward [45,46].

Phthalates may also play a role in the association between soft drinks consumption and fracture. Phthalates are widely used to make bottles for soft drinks. In animal studies, female rats treated with phthalates showed significant dose-dependent fetal skeletal malformations and bone homeostasis imbalances (e.g., deformities, delayed ossification, and skeletal variants) [47]. It can affect the actin cytoskeleton in Py1a osteoblasts and inhibits the calcium signaling pathways which are involved in bone proliferation, bone remodeling, and osteoblastic proliferation [48,49,50]. A study conducted in South Korea found that phthalates are directly associated with low bone mass and osteoporosis in women, regardless of calcium intake or physical activity [51].

There are some limitations in the current study. Firstly, the fracture was based on self-report. The fracture date was based on the participants’ recollection of the age at which the fracture occurred, which may be affected by recall bias. However, a large cohort study has shown that this approach is a feasible alternative [11,52]. Secondly, we did not have any objective measurement of bone health, such as BMD. Thirdly, the site of the fracture was not reported in this study. Fourthly, we did not have information of the types of soft drinks (i.e., carbonated soft drinks or non-carbonated soft-drinks). Thus, we could not assess the association between different types of soft drinks and fractures. Moreover, dietary data without including dietary supplements and bisphosphonates (drugs for osteoporosis) might overestimate the link between soft drinks and bone fractures [53]. Finally, soft drinks are a component of the modern dietary pattern. The observed findings may be an indicator of an overall effect of the modern dietary pattern and sedentary lifestyle. Although we adjusted for dietary patterns and physical activity, residual confounding is possible.

## 5. Conclusions

In summary, a high consumption of soft drinks is associated with fracture risk. Daily soft drinks consumption was associated with a doubled risk of fracture independently of sociodemographic factors and overall dietary patterns. This study highlights the important role of soft drinks in fracture risk among adults. To increase or maintain bone mass and reduce the risk of fracture, public health and clinical interventions should take into account reducing soft drinks consumption as an important strategy for the individual and population levels.

## Figures and Tables

**Table 1:** Sample characteristics in 2004 by soft drinks consumption.

	None	<1 Time/Week	1–2 Time/Week	3–4 Time/Week	Almost Daily
	N = 6822	N = 1363	N = 544	N = 156	N = 101
Age (years)	48.5 (13.1)	43.5 (13.4)	38.8 (12.4)	37.6 (14.1)	41.2 (14.3)
Sex					
Men	3452 (50.6%)	534 (39.2%)	226 (41.5%)	68 (43.6%)	66 (65.3%)
Women	3370 (49.4%)	829 (60.8%)	318 (58.5%)	88 (56.4%)	35 (34.7%)
Education					
Low	3077 (45.2%)	461 (33.9%)	103 (18.9%)	23 (14.8%)	32 (31.7%)
Medium	2243 (32.9%)	486 (35.8%)	213 (39.2%)	52 (33.5%)	34 (33.7%)
High	1489 (21.9%)	412 (30.3%)	228 (41.9%)	80 (51.6%)	35 (34.7%)
Urbanization					
Low	1974 (28.9%)	329 (24.1%)	99 (18.2%)	20 (12.8%)	11 (10.9%)
Medium	2055 (30.1%)	373 (27.4%)	124 (22.8%)	33 (21.2%)	28 (27.7%)
High	2793 (40.9%)	661 (48.5%)	321 (59.0%)	103 (66.0%)	62 (61.4%)
Smoking					
Non smoker	4433 (65.0%)	988 (72.5%)	394 (72.4%)	109 (69.9%)	47 (46.5%)
Former smokers	239 (3.5%)	49 (3.6%)	13 (2.4%)	3 (1.9%)	3 (3.0%)
Current smokers	2145 (31.5%)	325 (23.9%)	137 (25.2%)	44 (28.2%)	51 (50.5%)
Alcohol drinking	2259 (33.4%)	441 (32.6%)	182 (33.5%)	57 (37.0%)	45 (44.6%)
Physical activity (MET, hours/week)	123.6 (117.5)	119.0 (104.5)	123.5 (109.6)	109.6 (100.0)	139.1 (115.8)
BMI (kg/m^2^)	23.3 (3.4)	22.9 (3.2)	22.8 (3.4)	22.8 (3.5)	22.2 (2.7)
Energy intake (kcal/d)	2204.5 (677.9)	2241.3 (622.9)	2239.6 (656.8)	2161.3 (647.6)	2295.4 (690.0)
Fat intake (g/d)	67.9 (38.5)	73.3 (38.4)	77.2 (42.0)	76.1 (42.0)	78.3 (45.4)
Protein intake (g/d)	66.5 (25.4)	68.1 (22.9)	71.5 (23.5)	70.3 (23.9)	71.1 (26.0)
Carbohydrate intake (g/d)	325.0 (110.6)	323.9 (101.9)	311.6 (99.3)	293.9 (98.4)	324.8 (108.0)
Calcium intake (mg/d)	383.4 (319.1)	384.8 (270.0)	388.2 (223.2)	381.3 (319.7)	369.4 (211.7)
Phosphorus intake (mg/d)	1007.3 (445.2)	1000.3 (359.4)	1003.6 (321.0)	995.7 (321.6)	1014.7 (337.9)
Traditional southern dietary pattern score	−0.1 (1.0)	0.3 (0.9)	0.3 (0.9)	0.2 (0.9)	0.2 (0.8)
Modern dietary pattern score	−0.1 (0.9)	−0.0 (0.9)	0.3 (1.0)	0.4 (1.1)	0.0 (1.0)
Soft drinks consumption (liter/week)	0.0 (0.0−0.0)	0.2 (0.0−0.5)	1.0 (0.5−1.5)	1.5 (0.5−2.0)	1.0 (0.5−2.0)
Hypertension	1411 (21.9%)	206 (15.8%)	67 (12.9%)	20 (13.4%)	12 (12.4%)
Diabetes	120 (1.8%)	8 (0.6%)	1 (0.2%)	0 (0.0%)	0 (0.0%)
Fracture	297 (4.4%)	76 (5.6%)	25 (4.6%)	11 (7.1%)	2 (2.0%)

Notes: Data are presented as mean (SD) or median (IQR) for continuous measures, and n (%) for categorical measures.

**Table 2:** Odds ratio (95%CI) for fracture by soft drinks consumption levels among Chinese adults attending China Health and Nutrition Survey (n = 17,383).

	**Frequency of Soft Drinks Consumption**					**P for Trend**
	**None**	**<1 Time/Week**	**1–2 Time/Week**	**3–4 Time/Week**	**Almost Daily**	
Model 1	1.00	1.40 (1.18–1.65)	1.53 (1.18–1.99)	2.43 (1.62–3.63)	2.59 (1.55–4.33)	<0.001
Model 2	1.00	1.33 (1.10–1.60)	1.44 (1.08–1.92)	1.89 (1.20–2.97)	2.48 (1.42–4.32)	<0.001
Model 3	1.00	1.30 (1.08–1.57)	1.39 (1.04–1.85)	1.79 (1.13–2.81)	2.30 (1.32–4.01)	<0.001
Model 4	1.00	1.16 (0.94–1.44)	1.32 (0.95–1.84)	1.70 (0.99–2.92)	2.72 (1.45–5.09)	<0.001
	**Soft Drinks Consumption**					
	**None**	**<1 L/Week**	**≥1 L/Week**			
Model 1	1.00	1.38 (1.15–1.66)	1.67 (1.32–2.11)			<0.001
Model 2	1.00	1.36 (1.11–1.66)	1.45 (1.12–1.88)			<0.001
Model 3	1.00	1.33 (1.09–1.62)	1.40 (1.08–1.81)			0.001
Model 4	1.00	1.21 (0.97–1.53)	1.28 (0.95–1.74)			0.001

**Notes:** Model 1 adjusted for age, sex; Model 2 further adjusted for energy and fat intake, education, income (tertiles), urbanicity (tertiles), smoking, alcohol drink, physical activity (continuous, MET hour/week), and BMI (continuous); Model 3 further adjusted for dietary patterns derived from factor analysis; Model 4 further excluded those participated only in the wave of the survey.

**Table 3:** Hazard ratio (95%CI) for fracture by soft drinks consumption levels among Chinese adults attending China Health and Nutrition Survey (N = 9914).

	**Frequency of Soft Drinks Consumption**					***p* for Trend**
	**None**	**<1 Time/Week**	**1–2 Time/Week**	**3–4 Time/Week**	**Almost Daily**	
Number of participants	7228	1702	667	222	95	
Incident cases	397	104	39	13	16	
Person years	34,415	10,615	3359	1019	430	
Incident rate (per 1000)	11.5	9.8	11.6	12.8	37.2	
Model 1	1.00	0.92 (0.74–1.15)	1.19 (0.85–1.66)	1.41 (0.80–2.46)	3.87 (2.34–6.41)	0.001
Model 2	1.00	0.93 (0.73–1.19)	1.17 (0.82–1.68)	1.13 (0.58–2.21)	4.67 (2.79–7.80)	0.002
Model 3	1.00	0.92 (0.72–1.17)	1.17 (0.81–1.67)	1.13 (0.58–2.21)	4.69 (2.80–7.88)	0.002
	**Soft Drinks Consumption**					
	**None**	**<1 L/Week**	**≥1 L/Week**			
Number of participants	7667	1307	777			
Incident cases	423	84	50			
Person years	36,565	8250	4217			
Incident rate (per 1000)	11.6	10.2	11.9			
Model 1	1.00	0.92 (0.73–1.17)	1.20 (0.89–1.62)			0.518
Model 2	1.00	0.96 (0.75–1.24)	1.16 (0.83–1.61)			0.533
Model 3	1.00	0.96 (0.75–1.24)	1.16 (0.83–1.61)			0.569

Notes: Model 1 adjusted for age, sex; Model 2 further adjusted for energy and fat intake, education, income (tertiles), urbanicity (tertiles), smoking, alcohol drink, physical activity (MET, hours/week), and BMI; Model 3 further adjusted for dietary patterns; Among the 9914 participants free of fracture at baseline, 569 developed fracture during 49,838 person-year follow-up. The mean follow-up duration was 5.0 (SD 2.1) year.
